# Sleep deprivation and NLRP3 inflammasome: Is there a causal relationship?

**DOI:** 10.3389/fnins.2022.1018628

**Published:** 2022-12-22

**Authors:** Mohammad Amini, Zahra Yousefi, Sayed Soran Ghafori, Gholamreza Hassanzadeh

**Affiliations:** ^1^Department of Neuroscience and Addiction Studies, School of Advanced Technologies in Medicine, Tehran University of Medical Sciences, Tehran, Iran; ^2^School of Allied Medical Sciences, Shahroud University of Medical Sciences, Shahroud, Iran

**Keywords:** sleep, sleep deprivation, inflammation, inflammasome, neuroinflammation

## Abstract

In the modern era, sleep deprivation (SD) is one of the most common health problems that has a profound influence on an individual’s quality of life and overall health. Studies have identified the possibility that lack of sleep can stimulate inflammatory responses. NLRP3 inflammasome, a key component of the innate immune responses, initiates inflammatory responses by enhancing proinflammatory cytokine release and caspase-1-mediated pyroptosis. In this study, NLRP3 modification, its proinflammatory role, and potential targeted therapies were reviewed with regard to SD-induced outcomes. A growing body of evidence has showed the importance of the mechanistic connections between NLRP3 and the detrimental consequences of SD, but there is a need for more clinically relevant data. In animal research, (i) some animals show differential vulnerability to the effects of SD compared to humans. (ii) Additionally, the effects of sleep differ depending on the SD technique employed and the length of SD. Moreover, paying attention to the crosstalk of all the driving factors of NLRP3 inflammasome activation such as inflammatory responses, autonomic control, oxidative stress, and endothelial function is highly recommended. In conclusion, targeting NLRP3 inflammasome or its downstream pathways for therapy could be complicated due to the reciprocal and complex relationship of SD with NLRP3 inflammasome activation. However, additional research is required to support such a causal claim.

## Introduction

Sleep is a universal phenomenon of life and is critical for survival. It can be termed as a spontaneously reversible state of body and mind, distinguished by muted consciousness and lowered behavioral reactivity to external stimuli. It has also been shown that it plays important roles in almost every aspect of our health and wellbeing. Adequate sleep is necessary for proper functioning of the body, and inadequate sleep has important health consequences including cardiovascular diseases, neurological disorders, and even early mortality, which comprise a substantial portion of healthcare costs worldwide (Chattu et al., 2019).

Inflammation is an important innate immune response to potentially dangerous substances including pathogens, chemicals, and cell debris. Inflammatory responses play a critical role in protecting the body by detecting and removing injurious stimuli. Inflammation is intended to be beneficial to healthy tissues, but when it happens persistently, it will be detrimental. It is strongly related to various disorders including rheumatoid arthritis, hypertension, diabetes, neurological diseases, various infections, and some types of cancer (Amini et al., 2020b; Yamamoto et al., 2021; Yousefi et al., 2021, 2022).

Sleep deprivation (SD) is considered to be a result of lifestyle habits, neurological problems, and different sleep disorders such as insomnia and obstructive sleep apnea (OSA) (Amini et al., 2020a; Hanson and Huecker, 2021). SD, meaning insufficient and irregular sleep, has been associated with induction of inflammation in the entire body (Wang et al., 2021). Emerging evidence suggests that inflammation plays a key role in progression of SD-related impairments that are often not easily recognized. Thus, projects funded under this topic are highly encouraged. Despite decades of research, the mechanisms of the effects of SD on inflammation in the body have remained a mystery. Generally, insufficient sleep induces the release of important inflammatory cytokines such as pro-inflammatory cytokines tumor necrosis factor (TNF)-alpha, interleukin (IL)-1β, and IL-6 (Chennaoui et al., 2015). However, there are no specific inflammatory mediators that are involved in the response to sleep manipulation and its related mechanisms. This may partly be owing to the fact that SD-induced consequences are mostly reported in the central nervous system or are a result of some pre-existing inflammatory conditions such as obesity or type 2 diabetes mellitus. According to the results of recent investigations, inflammasomes can act as primary activators of inflammatory cytokines, thereby inducing inflammation. Inflammasomes are multimeric complexes of receptors and sensors of innate immune responses that through various mechanisms, play a critical role in inducing inflammatory responses against different pathogens (Noori et al., 2021). Although the actual role of inflammasomes in maintenance of immunologic homeostasis has not been clearly identified, they play an important role in clearing pathogens and damaged cells under physiological conditions. Whereas under pathological conditions, their overactivation may lead to disease onset or development (Höchsmann et al., 2019; Zhang H. et al., 2021). Nod-like receptor family pyrin domain-containing 3 (NLRP3) inflammasome is the most well-documented inflammasome type. The aberrant expression of NLRP3 inflammasome plays an important role in a wide range of disorders (Jourdan et al., 2013; Fusco et al., 2020). Recently, an increasing amount of data has demonstrated that activation of NLRP3 inflammasome is a critical mechanism in sleep modulation (Zielinski et al., 2017). In this review, light will be shed on the recent progress made in understanding the activation of NLRP3 inflammasomes and its potential therapeutic role in SD-induced inflammation, as it may be effective in treatment of a broad range of SD-induced health effects.

## Neuronal mechanisms of sleep

**FIGURE 1:**
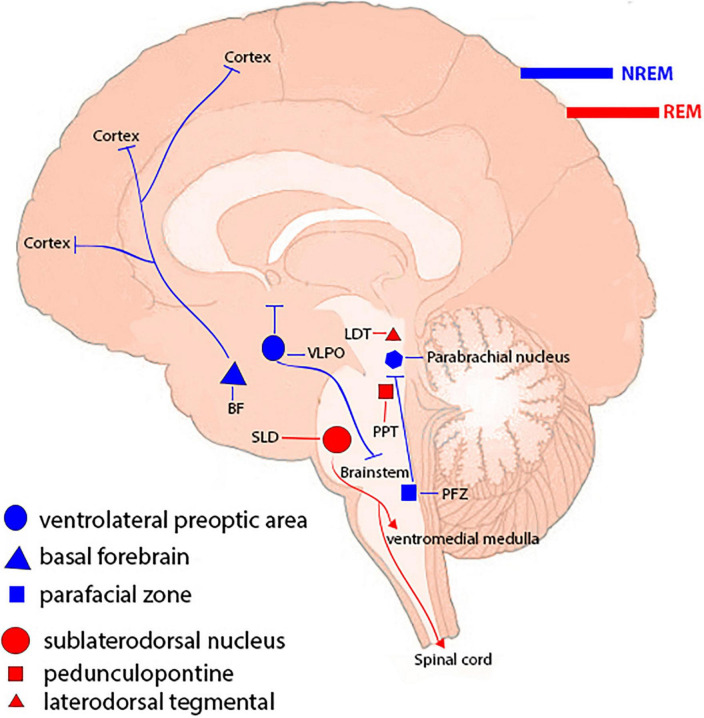
Sleep-controlling neural networks. Different types of sleep are attributed to a brain-wide neural network. Non-REM (NREM) sleep-promoting centers include the ventrolateral preoptic region, basal forebrain regions, the parafacial zone, and cortical sleep-active neurons. Neuronal networks in the brainstem, including the sublaterodorsal nucleus (SLD), pedunculopontine nucleus (PPT), and laterodorsal tegmental nucleus (LDTg), are involved in inducing rapid eye movement (REM) sleep.

Sleep is a common physiological behavior in almost all creatures and it accounts for approximately one-third of a human’s life, and lack of sleep has major clinical consequences. There are two forms of sleep: rapid eye movement (REM) sleep and non-REM (NREM) sleep (which has three stages). NREM and REM types of sleep alternate repeatedly during the night (Amini et al., 2020a). Different brain-wide neural networks are active during these two forms of sleep. Sleep normally starts with a NREM sleep stage, progresses through three NREM periods, and continues with a REM period. There are several NREM-promoting centers and they act as follows: The ventrolateral preoptic area (VLPO) GABAergic neurons, by inhibiting wake-promoting neurons in the hypothalamus and brainstem, the basal forebrain areas *via* ascending projections toward the cortex, the GABAergic parafacial zone by inhibiting the parabrachial nucleus, and the scattered cortical sleep-active neurons that contain both GABA and neuronal nitric oxide synthase. The neural networks in the pons are required for induction of REM sleep. Glutamatergic neurons in the sublaterodorsal nucleus (SLD) stimulate spinal inhibitory interneurons (GABAergic/glycinergic neurons) in the ventromedial medulla and spinal cord, resulting in motor neuron hyperpolarization and muscle tone reduction. Cholinergic pedunculopontine (PPT) and laterodorsal tegmental nucleus (LDTg) neurons also induce REM sleep by sending projections to the thalamus, whereas aminergic neurons block them during awakening and NREM sleep (España, 2017; Scammell et al., 2017; Figure 1).

## Circadian regulation of sleep

A biological clock is an internal mechanism that synchronizes the timing of internal rhythms with external light. The hypothalamus regulates it through the suprachiasmatic nucleus, which gets sensory input from the retinohypothalamic tract (RHT) based on luminance detected by the retina. Small projections from the RHT may also have an effect on the activity of sleep-promoting neurons in the preoptic region. Sleep pressure (extended intervals of alertness) drives the body to sleep when a certain amount of time has passed and regulates sleep intensity. NREM sleep-promoting substances (somnogens) such as adenosine (AD), prostaglandin D2, and cytokines such as IL-1 and TNF-α are most likely involved in this sleep/wake homeostatic response. The interplay of the circadian rhythm and sleep homeostasis can regulate timing, depth, and duration of sleep. In fact, the alignment between these two processes is crucial for the construction, maintenance, and function of the body and the brain (Deboer, 2018; Christensen et al., 2019). In addition, SD has been associated with many physiological and psychological disorders. For example, in healthy individuals, SD has been linked to diabetes type 2 and obesity, increased risk of cardiovascular diseases, and impairment of the functions of the immune system (Antza et al., 2022).

## Sleep deprivation epidemiology

Sleep deficiency can occur due to various circumstances and a broad spectrum of pathophysiological reasons including medication, debilitating diseases, neurological disorders, and sleep disorders. It is not clear how much an adult needs sleep, but it is considered to be 7–9 h per day. The American Academy of Sleep Medicine (AASM) and the Sleep Research Society (SRS) have stated that adults sleep less than 8 h a night (Chattu et al., 2019). As it appears, over a third of the U.S. citizens do not get enough sleep, as revealed in the latest survey by the Centers for Disease Control and Prevention (CDC) (Chattu et al., 2019). The U.S. National Academy of Medicine estimates that hundreds of billions of dollars are spent each year caring for people with sleep disturbances. For instance, one fifth of all the damage caused by major car collisions is attributed to drowsy driving (Higgins et al., 2017; Tefft, 2018). However, lack of sleep is not a concern for only the United States; it also affects other developing as well as developed countries such as the United Kingdom, the Netherlands, and Canada. According to a comprehensive review of the available literature, factors such as age, gender, race/ethnicity, and climatic conditions are the most common determinants that affect the prevalence of SD (Patel et al., 2010; Grandner et al., 2016). A study in Saudi Arabia showed that sleep loss is quite common among adolescents, with a higher incidence on weekdays (46%) than weekends (33%) (Nasim et al., 2019). In another study conducted in four cities in China, the incidence of insomnia was recorded at 37.75%, and less than 11.1% of the individuals suffering from insomnia took sleep medications on a regular schedule (Wang et al., 2016). It has transpired that adolescents who do not achieve adequate sleep are tend to gain weight primarily because of physical inactivity, they may suffer from depressive symptoms, take part in dangerous practices (i.e., drinking, smoking, drug use, etc.), and struggle with school (Pasch et al., 2010; Chattu et al., 2018). Finally, SD is a serious health problem due to its overall deleterious effects on the body. Given the evident implications of SD, tools for preventing and controlling it are highly demanded.

## Prevention and control of sleep deprivation

Overlooking the long-term aggregated impact of sleep restriction can accelerate the development of metabolic syndrome, cardiovascular diseases, diabetes, stroke, etc. (García-Aviles et al., 2021). A broad range of non-pharmacological and pharmacological therapies are available for patients with SD. Non-pharmacological approaches generate the most valid, reliable, and sustainable clinical advantages with the lowest levels of cost and complications. The first-line and best option for treatment of insufficient sleep is prevention by different mechanisms including creating a consistent schedule, avoiding caffeine and alcohol, having a bedtime ritual, and unplugging all extraneous electrical equipment. Pushing back school start times and increasing public awareness of the importance of adequate sleep and the consequences of SD, as well as sleep hygiene education programs are some examples of dealing with this problem. However, pharmacological approaches should be introduced as an alternative treatment option to improve sleep in patients when non-pharmacological interventions are ineffective or have failed. The primary treatment plans of sleep pharmacotherapy improve waking functions by improving sleep or by increasing energy during wakefulness. Alertness-promoting substances include amphetamine derivatives, modafinil, and caffeine. The most commonly-reported sleep-promoting substances are melatonin and zolpidem (Proctor and Bianchi, 2012; MacLeod et al., 2018; Jameie et al., 2019; Brito et al., 2020). Using medications to initiate extremely long periods of wakefulness could promote deleterious health consequences. Besides, frequent and patterned use of the medications stated here may actually result in creation of various problems such as physical or psychological dependency (De Matos et al., 2017). In general, a consistent schedule is a major element in this process, as is the cautious application of pharmacological drugs that improve sleep by enhancing alertness or aiding sleep (MacLeod et al., 2018; Jameie et al., 2019).

## The NLRP3 inflammasome signaling pathway

**FIGURE 2:**
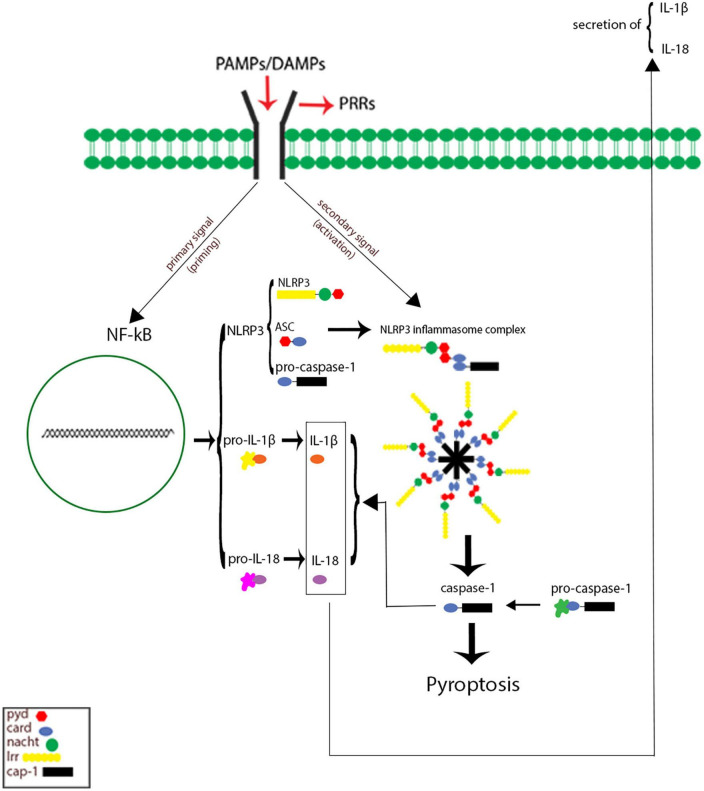
Signaling pathways of Nod-like receptor family pyrin domain-containing 3 (NLRP3) inflammasome. The first signal (priming: left) initiated through engagement of pathogen-associated molecular pattern (PAMPs) and danger-associated molecular patterns (DAMPs) with different pattern recognition receptors (PRRs), which in turn leading to activation of nuclear factor κB (NF-κB) and gene transcriptional of NLRP3 components, and pro inflammatory cytokines pro-IL-1 and pro-IL-18. The secondary signal (activation: right) is provided by a wide range of PAMPs and DAMPs that leading to formation of NLRP3 inflammasome complex. This complex activates caspase 1 enzyme, which resulting cleaves and secretion of activate forms of pro-IL-1 and pro-IL-18 as well as inducing cell death pyroptosis.

**FIGURE 3:**
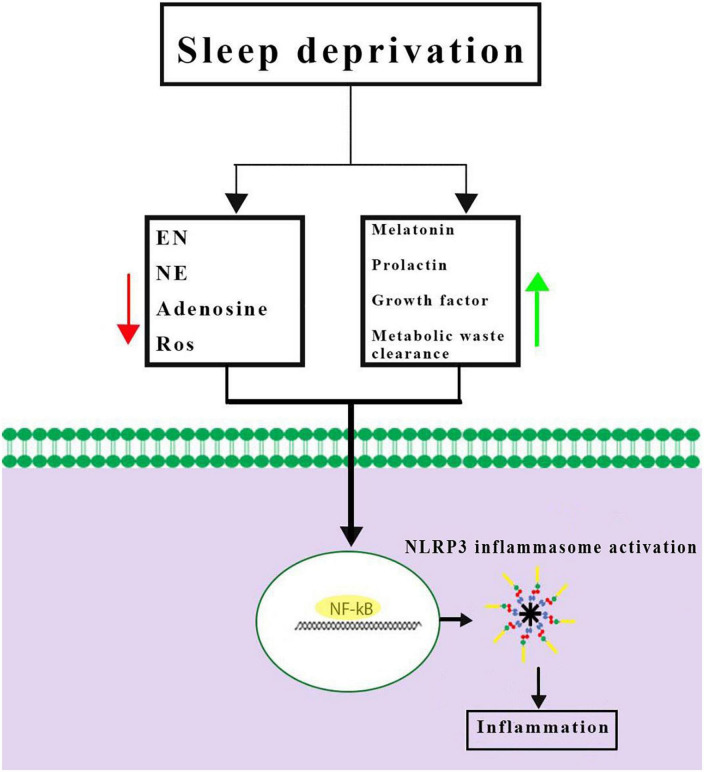
Possible mechanisms of Nod-like receptor family pyrin domain-containing 3 (NLRP3) inflammasome activation caused by sleep deprivation. Sleep deprivation can change neuromodulators and neuro-endocrines involved in sleep regulation and they could be altered during and after sleep deprivation. Together, these results demonstrated a priming signal for NLRP3 inflammasome activation by activating the NF-κB signaling pathway. EN, epinephrine; NE, norepinephrine; ROS, reactive oxygen species.

Inflammasomes are the most important components of innate inflammatory responses to harmful irritants. Once activated, they cause a rapid and highly inducible proinflammatory response. There are four types of inflammasomes: absentinmelanoma2 (Aim2), nucleotide-binding domain leucine-rich repeat-containing receptor 1 (thepyrin domain-containing NLRP1), NLRP3, and Nod-like receptor CARD domain-containing 4 (NLRC4). Although different inflammasomes have been identified, NLRP3 is one of the most well-characterized inflammasomes (Bazrafkan et al., 2018). In this section, the mechanisms that lead to activation of NLRP3 inflammasome are introduced and discussed. NLRP3 is composed of nucleotide-binding oligomerization domain (NACHT), apoptosis-speck-like protein (ASC), and procaspase-1 protein. NLRP3 inflammasome is mainly activated by pathogen-associated molecular patterns (PAMPs) and danger-associated molecular patterns (DAMPs). It has been revealed that two signals are involved in activation of NLRP3 inflammasome. The first signal (priming) is provided by different cytokines or engagement of various PAMPs and DAMPs with pattern recognition receptors (PRRs) that lead to activation of nuclear factor-κB (NF-κB) and transcriptional upregulation of NLRP3 components, pro-IL-18, and pro-IL-1β. In addition, NF-κB activation minimizes the activation threshold of NLRP3 inflammasome through additional post-translational modification (PTMs) (Yang et al., 2019; Zhao and Zhao, 2020). The second signal (activation) is initiated by a wide range of stimulations that appear during tissue damage, infections, etc., resulting in NLRP3 activation and formation of an inflammasome complex. Formation of NLRP3 complex results in activation of caspase 1, which induces the secretion of the activated form of pro-IL-1β and pro-IL-18 cytokines and development of inflammatory responses. Release of these cytokines plays a critical role in initiation of the host defense pathways and eradication of various pathogens. In addition, activation of inflammasomes leads to induction of an inflammatory form of cell death called pyroptosis (Paik et al., 2021; Figure 2). Previous studies have revealed that multiple brain-based immune proteins may contribute in sleep regulation by means of production of cytokines, phagocytosis or response to pathogens, as well as mRNA expression of the components of the NLRP3 inflammasome. According to a growing body of research, NLRP3 inflammasome plays a critical role in spontaneous sleep and sleep responses following sleep loss (Pourcet and Duez, 2020). It seems that the activity of NLRP3 inflammasome is a possible mechanism in sleep-modulation as well as increased cerebral blood flow (CBF) following SD, particularly in NREM sleep, as demonstrated in the electroencephalographic slow-wave activity (EEG SWA) (Zielinski et al., 2017, 2020). An effective activation of NLRP3 inflammasome is required for emergence of appropriate innate immune responses against various pathogens and prevention of catching or spreading pathogens. However, activation of these immune system proteins should be precisely controlled to avoid hyperinflammation or excessive deleterious effects (Zhang Y. et al., 2021). Recently, various studies have revealed a positive link between perturbation of the circadian clock and inflammation (Zielinski et al., 2020; Paik et al., 2021). Therefore, the underlying mechanisms and the related consequences remain a point of concern and should be the center of interest. SD leads to neuroendocrine dysregulation and may activate NLRP3 inflammasome (Figure 3).

## Role of NLRP3 inflammasome in SD-induced neuroinflammation

The innate immune system that uses inflammatory responses plays an important role in protection against various pathogens. The function of inflammation is eliminating and/or controlling damaged agents or the injurious components of tissues *via* phagocytosis as well as clearing them by inflammasomes (Evavold and Kagan, 2019). Neuroinflammatory response is a complex inflammatory response within the central nervous system. This inflammatory response is mediated by different cells such as microglia and astrocytes. Activation of glial cells leads to a variety of functions, including phagocytosis and release of some inflammatory mediators including cytokines, chemokines, reactive oxygen species, and secondary messengers (Brusaferri et al., 2022). SD can impair the normal functions of the nervous system, particularly brain functions such as memory, learning, decision making, and attention. In addition, SD has adverse effects on the rest of the body. For example, it impairs certain functions of the immune system and increases susceptibility to different infections. Over the last decade, neuroinflammation has been considered as a putative pathophysiological mechanism that contributes to the detrimental effects of sleep disturbance (Green et al., 2020). Mitogen-activated protein kinase (MAPK) signaling pathway plays an important role in transduction of extracellular signals to cellular responses, and it has been identified as a novel regulator of NLRP3 inflammasome activation. The stimulation of MAPK/NLRP3 axis in the hippocampus CA1 area, which is reversed by sleep recovery, is proposed as one of the mechanisms involved in this process (Fan et al., 2021). However, SD has been suggested to be an oxidative challenge for the brain. SD-induced oxidative stress can activate the NLRP3 inflammasome in rat hippocampus (Smith et al., 2021). In addition, previous research has shown that MCC950, as a potent and specific inhibitor of the NLRP3 inflammasome, can reverse SD-induced neuroinflammation and microglia activation by targeting NLRP3 (Smith et al., 2019). However, the precise mechanism by which SD causes neuroinflammation in the brain is not yet fully elucidated. There are many challenging questions that remain to be addressed. For instance, we know that insufficient sleep stimulates the NLRP3 inflammasome in neurons, microglia, and astrocytes. However, different effects have been observed depending on the involved portion of the brain (Niznikiewicz et al., 2017). The effects of NLRP3 inflammasome have been reported in the hippocampal region, but its effects have not yet been explored in other brain areas and thus remain unknown.

## SD-induced cognitive decline: Targeting NLRP3 inflammasome

People subjected to both total and partial SD, generally suffer from cognitive performance deficits as well as mood swings. Modafinil is a widely-used stimulant that was originally developed to treat paroxysmal narcolepsy. According to different research studies, modafinil improves NLRs inflammasome-mediated pyroptosis in mice with SD through increasing BDNF (brain-derived neurotrophic factor) stimulation in the hippocampus and synaptic plasticity, which in turn, improve learning, memory, and cognitive functions (Xiong et al., 2022).

## SD and depression: The role of NLRP3 inflammasome

The association between sleep and mental health disorders such as depression has been demonstrated through a number of research studies. Depression, as a common mood condition, is defined as feelings of unhappiness, loss, or rage that can interfere with daily activities and result in poor functional outcomes. It is believed that a total of 5% of adults experience depression. Poor sleep is both a potential risk and an indicator of depression. Studies have demonstrated that depression may be enhanced due to acute sleep deprivation or vice versa. However, the evidence is limited and conflicting, and more research is needed before any conclusions can be made. The P2 × 7 receptors are extracellular ATP-gated ion channels widely expressed in different cell types including blood, glial, neural, endothelial, muscle, and renal cells. These receptors also act as a second signal for NLRP3 inflammasome activation, resulting in the release of IL-1β and IL-18, and pyroptosis. Chronic SD may trigger depression by unbalancing neurochemicals such as serotonin in the brain. The likely cause for this is that ionotropic P2 × 7Rs increase the activation of NLRP3 inflammasome in the astrocytes, which result in development of depression-like behaviors caused by prolonged SD (6 h per day for 3–4 weeks). In addition, activated P2 × 7Rs reduce the expression of 5-HT2B receptors in astrocytes (Xia et al., 2020). Antidepressant fluoxetine can increase signal transducer and activator of transcription 3 (STAT3) activity (caused by the reduced expression of 5-HT2B receptors) while suppressing the activation of NLRP3 inflammasome and avoiding SD-induced neurotoxicity (Xia et al., 2017). Moreover, it is yet unclear if fluoxetine can improve sleep quality and minimize the negative effects of SD.

## SD-induced anxiety: Effects of NLRP3 inflammasome inhibition

This idea that an immunological dysfunction plays a significant role in mental health dates back many decades. SD is linked to a variety of mood disorders, including depression and anxiety. It has been revealed that long lasting SD enhances the circulating concentrations of inflammatory factors, which are known as risk factors of anxiety development and progression (Manchanda et al., 2018). A new study has found that injection of flavanol-rich dietary preparation (FDP) to mice following persistent SD exposure results in anti-anxiety activity by pharmacological inhibition of microglia activation and NLRP3 inflammasome activity. Moreover, release of IL-1 is inhibited by FDP-derived metabolites, and NLRP3-deficient animals show anxiety reduction in response to prolonged sleep deprivation. These results indicate that FDP’s anxiolytic effects are partly attributable to inhibition of NLRP3 activity. The same study also demonstrated that chronic sleep deprivation can alter the expression of circadian clock genes such as Bmal1 in the hippocampus, which plays a critical role in suppressing the expression of NLRP3 genes (Smith et al., 2021). These findings may explain how NLRP3 inflammasome is involved in neural networks and cognitive function pathways.

## Insomnia and NLRP3 inflammasome

Insomnia is described as having trouble falling asleep and staying asleep, or waking up too early in the morning. These sleep symptoms are accompanied with daily cognitive dysfunction including poor concentration, memory problems, learning disability, mood fluctuations, and so on. To be a person diagnosed with the insomnia disorder, a patient would have to report experiencing these symptoms at least three times a week and for a minimum period of 3 months. Insomnia is a relatively prevalent sleep disorder with a total population-based frequency of about 10% that is characterized with multiple etiologies as well as several subtypes (Rosenberg et al., 2021). Among different subtypes, the insomnia with objective short sleep duration (IOSSD) seems to be rapidly rising in recent years. The objective short sleep duration is commonly described as a continuous nocturnal sleep period of less than 6 h, as recorded by objective measurement techniques like polysomnography (PSG) (Fernandez-Mendoza et al., 2021). The latest research studies have demonstrated that chronic insomniacs are identified with overexpression of NLRP3, ASC, and caspase-1 in peripheral blood mononuclear cells (PBMCs) compared to controls. In addition, increased concentrations of NLRP3 plasma and activity of hypothalamic-pituitary-adrenal (HPA) axis are related to sympathetic hyperactivity. Epinephrine and norepinephrine are important catecholamines in the neuroendocrine system when sympathetic nervous system activity is dominant. A previous study demonstrated the activation of NLRP3 inflammasome in IOSSD patients and proposed that changes in epinephrine and norepinephrine levels in patients may be partly responsible for NLRP3 inflammasome activation *via* stimulation of leukocyte adrenergic receptors [e.g., Adrenoceptor Beta 2 (ADBR2) and Toll-like-receptor-4 (TLR-4)] and activation of NF-κB-mediated inflammatory signal pathway. In addition, studies have discovered that NLRP3 activity is positively related to short sleep duration and sleep fragmentation (Wang, 2020). In chronic insomnia patients, there is controversy in the findings that show the existence of a link between activation of NLRP3 inflammasome pathway and REM sleep duration (Wang et al., 2020; Aghelan et al., 2022).

## Sleep apnea syndrome and NLRP3 inflammasome

Sleep apnea syndrome is defined by episodes of breathing interruption during sleep, and the air-flow stoppage can last for over 10 s. There are three categories of sleep apnea called central, complex, and obstructive. Central sleep apnea happens when cessation in breathing occurs mainly due to lack of central nervous system’s drive to the muscles to breathe during sleep. Complex sleep apnea is a combination of obstructive and central sleep apneas (Orr et al., 2017). Obstructive sleep apnea (OSA) is the most frequently occurring form, affecting approximately 14% of males and 5% of females and its highest prevalence is reported among middle-aged males. It occurs when complete or partial airway obstruction, caused by pharyngeal collapse during sleep, results in loud snoring or choking, intermittent hypoxemia, sleep fragmentation, and excessive daytime sleepiness (Osman et al., 2018). Recent studies have shown that OSA may increase the incidence of chronic kidney disease and acute kidney injuries. However, despite various undesirable side effects including dry mouth, increased number of awakenings, blocked-up nose, mask pressure, and mask leaks, continuous positive airway pressure (CPAP) is the most widely used therapy for this sleep disorder. Currently, no effective strategy is available for prevention of the renal injuries caused by OSA (Wang et al., 2020; Fernandez-Mendoza et al., 2021). One study indicated that pharmacological inhibition of mammalian target of rapamycin (mTOR)/NLRP3 axis by rapamycin, alleviates the OSA-induced renal damage (Liu et al., 2022). OSA-related type-2 diabetes mellitus (T2DM) is another frequent co-morbidity that affects nearly half of the adult patients and is associated with increased morbidity, mortality, and healthcare costs (Jehan et al., 2018). In recent years, microRNAs (miRNAs) and long non-coding RNAs (lncRNAs) such as metastasis-associated lung adenocarcinoma transcript 1 (MALAT1) have been proposed as new and crucial regulators of diverse biological processes (Vafadar et al., 2019). Mechanistically, it has been shown that NLRP3 inflammasome-mediated inflammatory responses are activated in T2DM animals with OSA as well as cell models, which may be related to MALT1 overexpression leading to inhibition of miR-224-5p in the hippocampus (Du et al., 2020). Most of the studies have focused on the intermittent hypoxemia and are limited by the absence of other cardinal features of OSA, including sleep fragmentation. In other studies investigating plasma NLRP3 levels in OSA patients, it has been observed that the levels of proinflammatory cytokines IL-1 and IL-18 are increased independent of NLRP3 levels, and this controversy remains to be elucidated (Kerget et al., 2021).

The take-home message of this research is that: (i) increased activity of the sympathetic nervous system and increased oxidative stress are considered as primary activators of NLRP3 activation, which in turn, play an essential role in overproduction of potent proinflammatory cytokines, particularly IL-1 and IL-18. (ii) Knockout of NLRP3 genes or pharmacological blockage can alleviate OSA-associated neurocognitive impairment, pulmonary hypertension, cardiac injury, T2DM, and renal injury.

## Traumatic brain injury-induced sleep disturbance: Role of NLRP3 inflammasome

Sleep disturbance after traumatic brain injury (TBI), also known as intracranial injury, occurs when a sudden trauma damages the areas of the brain involved with controlling sleep patterns (Jehan et al., 2018; Liu et al., 2022). Sleep dysregulation is frequent after TBI, and approximately 30–70% of the patients experience sleep disturbances such as insomnia, daytime fatigue, and disruptions in their sleep-wake cycle (Vafadar et al., 2019; Du et al., 2020). Recent findings from human and rodent studies have indicated an upregulation in NLRP3-related molecules following TBI (Grima et al., 2016; Ismael et al., 2021). Other findings have suggested that NLRP3 inflammasomes contribute to deregulated sleep that occurs acutely or more persistently after TBI (Zielinski et al., 2021). A variety of rodent model studies have indicated that selectively suppressing the NLRP3 inflammasome can mitigate neuroinflammation and improve outcomes following TBI (Sandsmark et al., 2017; Mohammed et al., 2020). The studies included in the present review are summarized in Table 1.

**TABLE 1:** Summary table of preclinical and clinical studies included in the present review study.

#	Findings	References
**Preclinical studies**		
1	The activation of the NLRP3 inflammasome can modulate sleep induced by wakefulness. These findings show that the NLRP3 inflammasome is an important mechanism involved in sleep responses to sleep loss and pathogen components in the brain.	(Zielinski et al., 2017)
2	The biological clock regulates NLRP3 expression and activation in diverse tissues. Circadian oscillations of NLRP3 signaling is lost in clock disruption models, which contributes to the development of disorders.	(Pourcet and Duez, 2020)
3	NLRP3 inflammasomes are involved in neurovascular coupling involving SWA.	(Zielinski et al., 2020)
4	The MAPK/NLRP3 axis may be important in the development of SD neuronal pyroptosis. The NLRP3 inflammasome is thought to be a potential therapeutic target for SD-induced neuroinflammation in the hippocampus.	(Fan et al., 2021)
5	SD modifies the expression of the circadian gene Bmal1, which controls NLRP3 expression and IL-1 production. FDP’s anxiolytic effects may be mediated by inhibiting NLRP3 inflammasome activity.	(Smith et al., 2021)
6	The administration of the NLRP3 inhibitor (MCC950) prevents SD-induced changes in microglia morphology.	(Smith et al., 2019)
7	SD activates the NLRP3 inflammasome in neurons, astrocytes, and microglia, although this activation varies depending upon the brain area.	(Niznikiewicz et al., 2017)
8	Treatment with modafinil reduced inflammasome activity and neuronal pyroptosis *via* the NLRP3/NLRP1/NLRC4-caspase-1-IL-1β pathway. Targeting the regulation of impaired neuronal pyroptosis and neuroinflammation may be a promising therapeutic strategy for treatment of SD.	(Xiong et al., 2022)
9	The P2 × 7 receptors promote the formation of the NLRP3 inflammasome and the ATP-induced release of mature interleukin (IL)-1b and IL-18 from astrocytes, which leads to the development of chronic SD-induced depressive-like behavior.	(Xia et al., 2020)
10	Fluoxetine can prevent the activation of NLRP3 inflammasome and avoiding SD-induced neurotoxicity. Furthermore, the activation of STAT3 is an important target that regulated the expression of NLRP3 inflammasomes in an SD model.	(Xia et al., 2017)
11	This study identified the effects of rapamycin on OSA-associated renal injury. Inhibiting the mTOR signaling pathway by rapamycin can significantly reduce the levels of NLRP3 and organ damage caused by OSA.	(Liu et al., 2022)
12	MiR-224-5p reduces inflammation through the regulation of NLRP3 expression in T2DM With OSA, which finally regulated the NLRP3/IL1β pathway in the hippocampus.	(Du et al., 2020)
13	Mice lacking NLRP3 had attenuations in the significant increased amounts of NREM sleep and EEG delta power occurring 24 h after TBI and the significant reductions seen 2 months after TBI that were observed in wild-type mice. The findings suggest that NLRP3 inflammasomes contribute to dysregulated sleep occurring acutely or more persistently after TBI.	(Zielinski et al., 2021)
**Clinical studies**		
14	Sleep fragmentation may contribute to dysregulation of NLRP3 inflammasome in IOSSD. Potential mechanisms linking sleep loss and NLRP3 inflammasome may include the activation of sympathetic system, hypothalamus-pituitary-adrenal axis and production of ROS.	(Wang et al., 2020)
15	NLRP3 inflammasome gene expression have a negative correlation with REM sleep duration. This evidence suggest that NLRP3 inflammasome is involved in the pathogenesis of the sleep disorders. inhibitors of the NLRP3 inflammasome may be promising therapeutic agents in sleep deprivation and sleep fragmentation.	(Aghelan et al., 2022)
16	There was no difference in the OSA development according to NLRP3 level.	(Kerget et al., 2021)

## Conclusion and perspectives

In recent years, a growing body of evidence has emerged that demonstrates the importance of the mechanistic connections between NLRP3 and SD-induced detrimental consequences (Supplementary Figure 1), but there are still several pieces of the puzzle to be put in place. For ethical reasons, clinical studies cannot evaluate the expressions of NLRP3 inflammasome in the human brain, which can directly reflect the effects of sleep on neuroinflammation, and thus there is a need for more clinically relevant data. In animal research, issues and challenges can be seen from different viewpoints: (i) some animals show differential vulnerability to the effects of SD compared to humans. (ii) Additionally, the effects of sleep differ depending on the SD technique employed and the length of SD. Moreover, paying attention to the crosstalk of all the driving factors of NLRP3 inflammasome activation, such as inflammatory responses, autonomic control, oxidative stress, and endothelial function is highly recommended to prevent possible comorbidities, which are commonly seen in patients. In conclusion, NLRP3 inflammasome or its downstream pathways, is a potential target for therapy in order to improve the clinical outcomes of SD. However, the treatment process can be complicated due to the reciprocal and complex relationship of SD with NLRP3 inflammasome activation. Nonetheless, additional research is required to support such a causal claim and there are some factors to consider: First, why sleep deprivation occurs, and second, how do different types of SD (Total, REM, and NREM) contribute to the NLRP3 inflammasome activity.

## Author contributions

MA wrote the original text, revised, and assisted with editing the manuscript. ZY helped generate ideas for the framework of the manuscript, conducted the linguistic sorting investigation, and assisted with editing the manuscript. SG drew the figures and assisted with revising the manuscript. GH helped generate ideas for the framework of the manuscript and revised the manuscript. All authors contributed to and approved the final manuscript.

## Supplementary material

Supplementary Figure 1 Summary of the relationship between sleep deprivation (SD) and Nod-like receptor family pyrin domain-containing 3 (NLRP3) inflammasome.
