# Supplementary figures and images for: Sleep deprivation and NLRP3 inflammasome: Is there a causal relationship?
Source: Front Neurosci. 2022 Dec 22;16:1018628. doi: 10.3389/fnins.2022.1018628 (PMC9815451; doi:10.3389/fnins.2022.1018628)

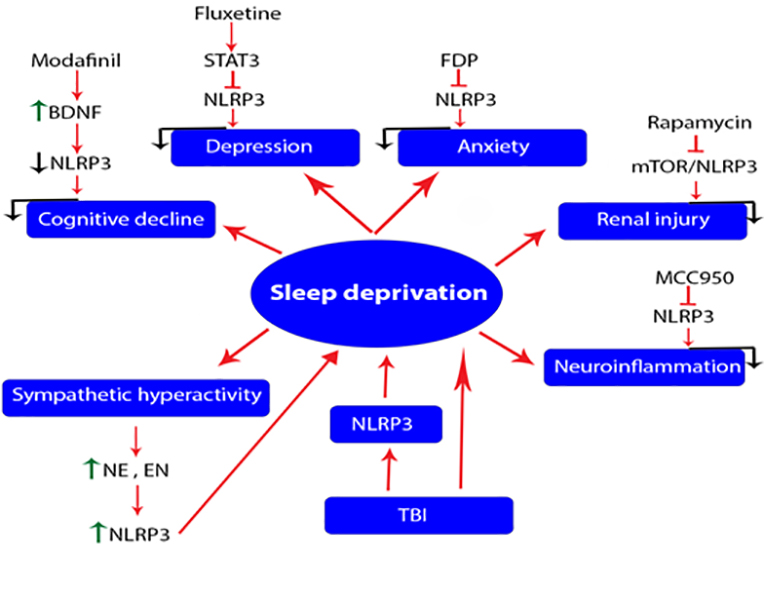

Supplement: Supplementary Figure 1 — Summary of the relationship between sleep deprivation (SD) and Nod-like receptor family pyrin domain-containing 3 (NLRP3) inflammasome. [file Image_1.JPEG]
